# Proopiomelanocortin, glucocorticoid, and CRH receptor expression in human ACTH-secreting pituitary adenomas

**DOI:** 10.1007/s12020-016-0990-x

**Published:** 2016-05-24

**Authors:** Maria Francesca Cassarino, Antonella Sesta, Luca Pagliardini, Marco Losa, Giovanni Lasio, Francesco Cavagnini, Francesca Pecori Giraldi

**Affiliations:** 1 Neuroendocrinology Research Laboratory, Istituto Auxologico Italiano IRCCS, Cusano Milanino, Milan, Italy; 2 Department of Neurosurgery, Ospedale San Raffaele IRCCS, Milan, Italy; 3 Department of Neurosurgery, Istituto Clinico Humanitas, Rozzano, Milan, Italy; 4 Department of Clinical Sciences and Community Health, University of Milan, Piazzale Brescia 20, 20149 Milan, Italy

**Keywords:** Cushing’s disease, POMC, ACTH, CRH, Dexamethasone

## Abstract

ACTH-secreting pituitary tumors are by definition partially autonomous, i.e., secrete ACTH independent of physiological control. However, only few, small-sized studies on proopiomelanocortin (*POMC*) and its regulation by corticotropin-releasing hormone (CRH) or glucocorticoids are available. Objective of the present study was to report on constitutive and CRH- and dexamethasone-regulated *POMC*, *CRH* (*CRH*-*R1*), and glucocorticoid receptor (*NR3C1*) gene expression in a large series of human corticotrope adenomas. Fifty-three ACTH-secreting adenomas were incubated with 10 nM CRH or 10 nM dexamethasone for 24 h. *POMC*, *CRH*-*R1,* *NR3C1*, and its alpha and beta isoforms were quantified and medium ACTH measured. Constitutive *POMC* expression proved extremely variable, with macroadenomas exhibiting higher levels than microadenomas. *POMC* increased during CRH in most specimens; conversely, changes induced by dexamethasone were varied, ranging from decrease to paradoxical increase. No correlation between *POMC* and ACTH was detected in any experimental condition. *CRH*-*R1* expression was not linked to the response to CRH while *NR3C1* was expressed at greater levels in specimens who failed to inhibit during dexamethasone; glucocorticoid receptor α was the more abundant isoform and subject to down-regulation by dexamethasone. Our results demonstrate a considerable variability in *POMC* expression among tumors and no correlation between *POMC* and ACTH, suggesting that POMC peptide processing/transport plays a major role in modulating ACTH secretion. Further, *CRH*-*R1* and *NR3C1* expression were not linked to the expected ligand-induced outcome, indicating that receptor signaling rather than abundance determines corticotrope responses. Our findings pave the way to new avenues of research into Cushing’s disease pathophysiology.

**Electronic supplementary material:**

The online version of this article (doi:10.1007/s12020-016-0990-x) contains supplementary material, which is available to authorized users.

## Introduction

ACTH-secreting pituitary tumors are characterized by autonomous yet still responsive ACTH secretion. ACTH secretion is autonomous in as much as the tumoral corticotrope continues to secrete ACTH notwithstanding high cortisol levels, thus appears insensitive to physiological negative feedback. On the other hand, ACTH secretion by tumoral corticotropes is sensitive to stimulation by corticotropin-releasing hormone (CRH) and inhibition by high doses of synthetic steroids. Indeed, sensitivity to the two major regulators of the hypothalamo-pituitary-adrenal (HPA) axis is unique to pituitary ACTH-secreting tumors and allows the distinction from extrapituitary ACTH-secreting tumors [1].

Mechanisms underlying the preserved sensitivity to CRH and high doses of corticosteroids are as yet unknown. No mutation in the glucocorticoid receptor (GR) gene (*NR3C1*) has been detected except for anecdotal patients with large, invasive tumors [2]. Alterations in factors which modulate corticosteroid action on the proopiomelanocortin (*POMC*) promoter, e.g., Brg1 [3], or its intrapituitary availability, e.g., 11ßHSD [4] have been reported but appear variably linked to tumoral sensitivity to steroid-induced suppression. As regards the main pituitary CRH receptor isoform, CRH-R1, studies on a dozen of adenomatous specimens revealed wild-type sequence and over expression compared to normal pituitaries [5, 6]. Of note, current knowledge on tumoral corticotrope secretory pathophysiology is based upon studies performed on small series of specimens, a consequence of the rarity of Cushing’s disease and paucity of available surgical material. Indeed, *POMC* expression itself has been evaluated in studies reporting on 20 tumoral specimens at most [7, 8].

The aim of the present study was to expand upon our recent findings on a large series of ACTH-secreting pituitary adenomas [9]. In this study, we reported on the marked variability in ACTH secretion by human corticotrope tumors in vitro. Given this premise, we decided to evaluate *POMC* expression as well as *CRH*-*R1* and glucocorticoid receptor expression in ACTH-secreting pituitary adenomas and their response to CRH and dexamethasone in vitro.

## Materials and methods

### Patients

Fifty-three ACTH-secreting pituitary adenomas were collected during surgery and established in culture according to our usual protocol (see below). Diagnosis of Cushing’s disease had been established by standard diagnostic procedures [1, 10]. The cohort comprised 46 women and 7 men with overt pituitary-dependent hypercortisolism, 24 macroadenomas (i.e., >10 mm diameter) and 29 microadenomas (<10 mm diameter). Of note, the present series includes 33 adenomas from the previous study on ACTH secretion [9] and 20 additional specimens. None of the patients had been treated with cabergoline or pasireotide prior to surgery. As regards responses to dynamic tests, an increase in plasma ACTH by at least 30 % was considered a response to CRH testing whereas a decrease by at least 50 % in urinary or serum cortisol was considered a response to high-dose dexamethasone testing. The study was approved by the Ethical Committee of our Institution, and informed consent was obtained from patients at the referring neurosurgical centers. Surgical outcome was defined on clinical (e.g., development of adrenal insufficiency and/or requirement for steroid replacement therapy) and hormonal grounds (i.e., decrease of morning serum cortisol to below the normal range, normalization of urinary free cortisol) [11].

### Pituitary adenoma primary cultures

Tumoral specimens were dispersed by enzymatic digestion [9, 12, 13] and plated at 20 × 10^3^ to 300 × 10^3^ per well, according to specimen abundance. Cells were attached in DMEM, 10 % fetal calf serum and antibiotics for 3–5 days, then washed with DMEM and 0.1 % BSA prior to challenge with 10 nM CRH or 10 nM dexamethasone (all reagents from Sigma-Aldrich, St. Louis, MO, USA). Control wells were incubated with DMEM + 0.1 % BSA alone and each treatment was performed in triplicate or quadruplicate, depending upon cell abundance. After 24 h incubation, medium was collected for ACTH measurement [9] and cell RNA extracted (TRIzol reagent, Invitrogen, Milan, Italy).

### ACTH assays

ACTH was measured immunoradiometric assay (IRMA); the IRMA Advantage kit (Nichols Institute, San Juan Capistrano, CA, USA) was used from 2000 to 2005 (20 tumoral specimens) then with the kit (33 tumoral specimens) provided by Diasorin (Saluggia, Italy) according to manufacturers’ instructions; sensitivity and intraassay coefficient of variation are 1.5 pg/ml and 4.8 %, 1.2 pg/ml and 5.9 %, respectively, for Nichols Institute and Diasorin assays. All samples from a given specimen were measured in the same run. Only pituitary cultures secreting at least 10 pg/10^5^ cells ACTH were included in the study, in order to ensure the presence of tumoral corticotropes. Indeed, normal corticotropes are silenced by long-standing hypercortisolism in patients with Cushing’s disease as over 1 year is usually required for recovery of normal corticotropes [14] and reappearance of the ACTH response to CRH [15]. Within specimen variability of spontaneous ACTH secretion, calculated as the coefficient of variation (i.e., SD/mean), averaged 7 %; a difference of at least 20 % from control ACTH secretion, i.e., >twofold greater than the variability of spontaneous secretion, was taken to indicate a response [16]. The proportion of ACTH responders to CRH was 58 and 63 % among specimens collected prior to 2011 [9] and in the following 5 years, respectively. As regards the ACTH response to dexamethasone, inhibition was observed in 50 % of specimens from the 2011 series [9] and in 40 % of specimens collected thereafter.

### Quantitative real-time PCR

RNA (100 ng) was reverse-transcribed by Superscript Vilo cDNA synthesis Kit (Invitrogen, Milan, Italy) and quantitative Real-Time PCR performed on a 7900 HT sequence Detection System (Applied Biosystem, Foster City, CA, USA), using Platinum Quantitative PCR Supermix-UDG with premixed ROX. Taqman assay (Applied Biosystem, Foster City, CA, USA) was used for detection of the following genes: *POMC* (probe Hs00174947_m1), *CRH*-*R1* (probe Hs01062290_m1), *NR3C1* i.e., glucocorticoid receptor (probe Hs00230813_m1), *RPLP0* i.e., housekeeping gene (probe Hs99999902_m1). SYBR green assay (Applied Biosystem, Foster City, CA, USA) was used to discriminate between glucocorticoid receptor (GR) isoforms according to the procedure devised by Ma et al. [17]. The following primer pairs *GRα* forward CTATGCATGAAGTGGTTGAAAA and reverse TTTCAGCTAACATCTCGGG; *GRß* forward GAAGGAAACTCCAGCCAGAA and reverse CCACATAACATTTTCATGCATAGA, both normalized to *GAPDH* (forward GGACCTGACCTGCCGTCTAG, reverse TAGCCCAGGATGCCCTTGAG) were used. The melting curve of PCR products allowed separation of genuine products from non-specific products and primer dimers. Basal expression data (2^−ΔCt^) were calculated and normalized to *RPLP0* or *GAPDH*, respectively, in order to normalize for cell abundance; expression after treatment was analyzed as 2^−ΔΔCt^ and expressed in fold increase. Of note, CRH or dexamethasone treatments did not affect housekeeping gene expression. Supplementary information lists the number of specimens available for each analysis.

### Statistical analysis

Data are expressed as mean ± SEM. Differences between groups were established by Wilcoxon signed rank test or Mann–Whitney test, as appropriate. Associations between variables were assessed by linear regression analysis. Significance was accepted for *p* values <0.05.

## Results

**Fig. 1:**
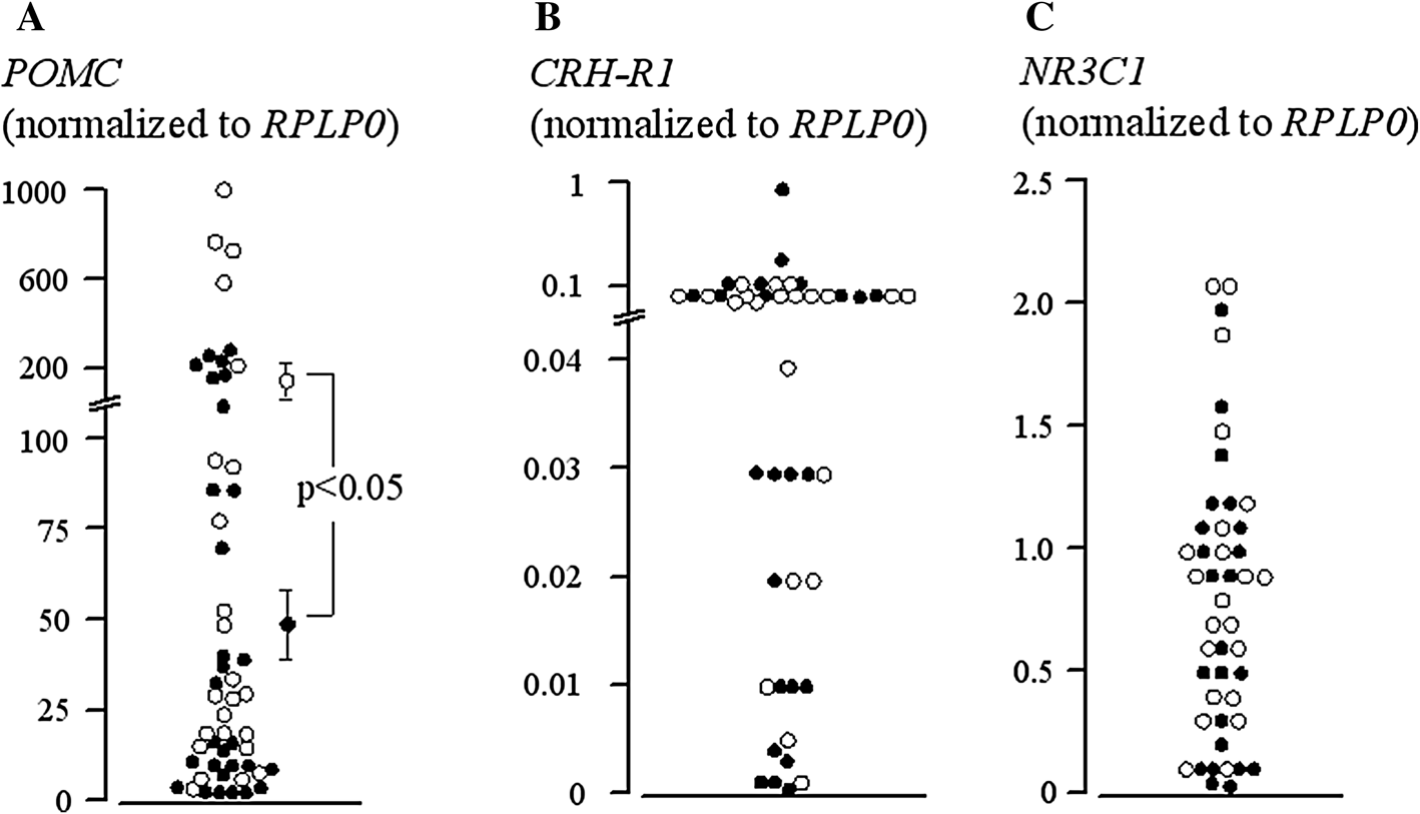
Constitutive expression of *POMC*, *CRH*-*R1* and *NR3C1* in individual human ACTH-secreting pituitary adenomas. *Empty circles* are macroadenomas, *filled circles* microadenomas

**Fig. 2:**
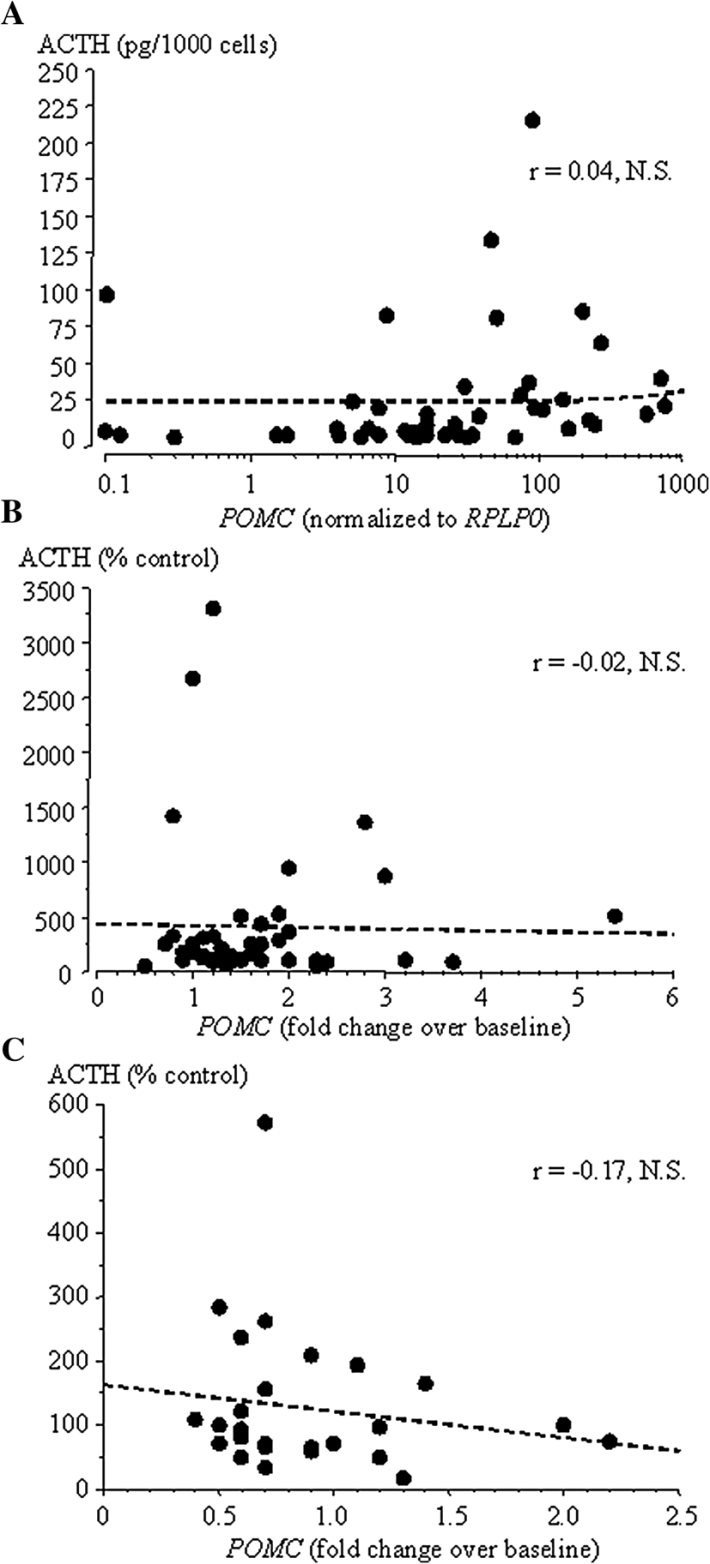
Correlation between *POMC* and ACTH in human ACTH-secreting pituitary adenomas in unstimulated conditions (**a**), during CRH (**b**) and during dexamethasone (**c**)

Quantification of *POMC* mRNA revealed abundant gene expression and considerable variability among tumoral specimens with up to 100-fold greater expression in some specimens (Fig. 1a). Subgroup analysis showed that macroadenomas exhibit higher levels of *POMC* compared to microadenomas (180.6 ± 61.05 vs. 49.5 ± 14.62 normalized expression, *p* < 0.05) but none of the other variables, e.g., sex, age, and surgical outcome, proved a significant contributor to *POMC* variability. *POMC* expression was not correlated with ACTH secretion in the same specimen (*r* = 0.04, N.S.; Fig. 2a). Lastly, no difference in *POMC* expression was observed between specimens collected prior to 2011 [9] and thereafter (121.7 ± 42.69 vs. 94.5 ± 48.80 normalized expression, N.S.).

The *CRH*-*R1* receptor gene was expressed at variable levels in corticotrope tumors (Fig. 1b); no difference according to tumor size was observed (0.09 ± 0.01 vs. 0.08 ± 0.02 normalized expression for macro- and microadenomas, respectively, N.S.)

Expression of the glucocorticoid receptor gene was more abundant than *CRH*-*R1* (Fig. 1c) and evenly distributed among specimens. In fact, no differences according to tumor size or surgical outcome were observed (0.94 ± 0.13 vs. 0.68 ± 0.12 normalized expression for macro- and microadenomas, respectively, N.S; 0.87 ± 0.11 vs. 0.67 ± 0.15 normalized expression for cured and uncured, respectively, N.S.). As regards GR isoforms, GRα was markedly more abundant than GRß; in fact the latter was undetectable in over 50 % of specimens (36.9 ± 6.31 vs. 0.04 ± 0.01 normalized expression for GRα and GRß, respectively, *p* < 0.05). The GRα:GRß ratio ranged from 1000 to 1.000.000. No differences between GR isoforms were observed as regards tumor size or surgical outcome.

**Fig. 3:**
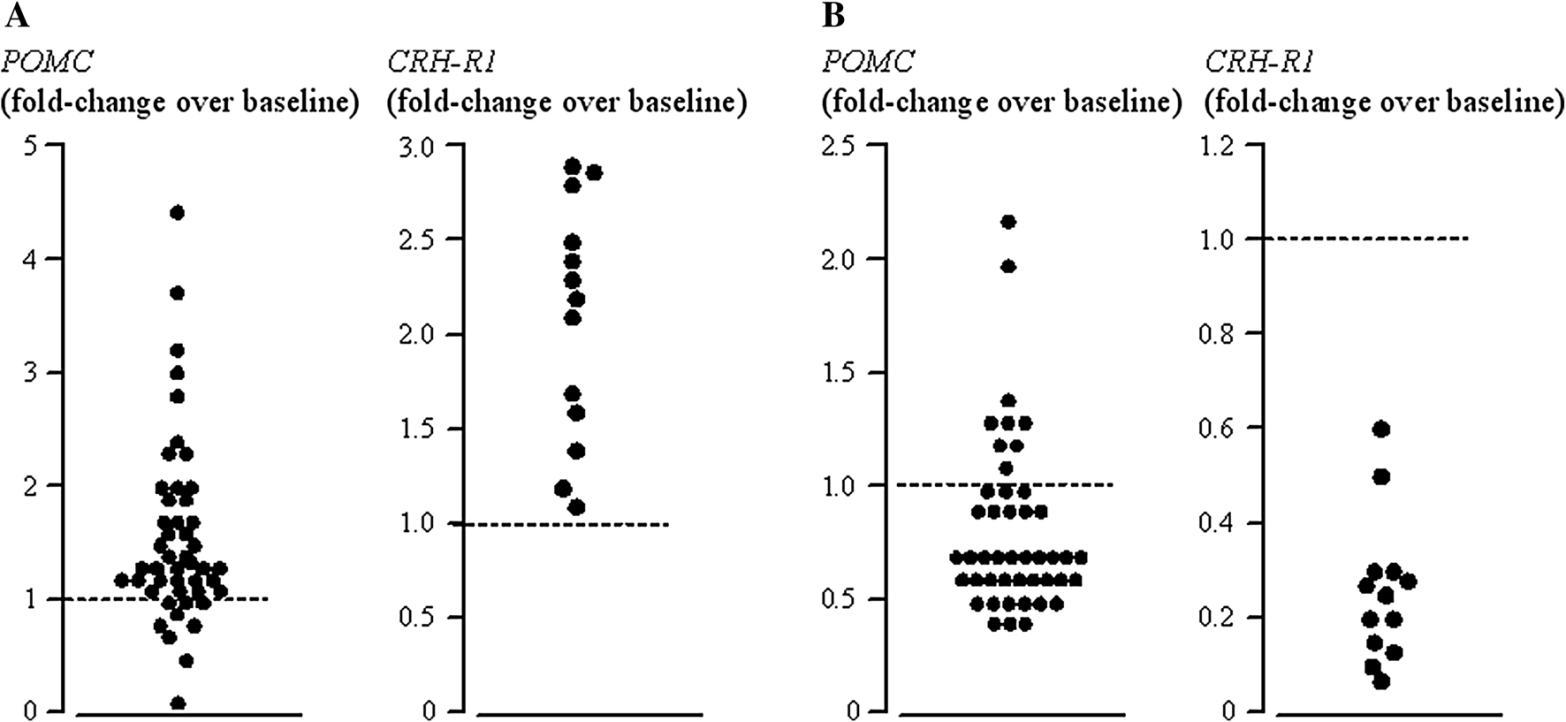
*POMC* and *CRH*-*R1* expression during incubation with 10 nM CRH (**a**) or 10 nM dexamethasone (**b**) in individual human ACTH-secreting pituitary adenomas. *Dashed line* represents baseline expression

Upon incubation with CRH, *POMC* increased markedly (1.63 ± 0.13 over baseline, *p* < 0.0001), as shown in Fig. 3a. Of interest, the *POMC* and ACTH responses to CRH were not correlated with each other (*r* = −0.02, N.S.; Fig. 2b) nor was the increase in *POMC* more pronounced in patients who responded to CRH stimulation prior to surgery (1.65 ± 0.35 vs. 1.99 ± 0.29 over baseline, for in vivo CRH responders and non-responders, respectively, N.S.). Overall, the in vitro *POMC* response pattern concurred in the in vivo CRH response pattern in 60 % of patients. There was no significant association between either response and CRH receptor abundance (*r* = 0.24, N.S. for *POMC* and *CRH*-*R1* and *r* = 0.2, N.S. for ACTH and *CRH*-*R1*). Indeed, *CRH*-*R1* levels were comparable among CRH responders and non-responders (0.09 ± 0.01 vs. 0.07 ± 0.03 normalized expression, N.S.). An increase in *CRH*-*R1* was observed during incubation with CRH (2.08 ± 0.29 over baseline, *p* < 0.05), whereas *CRH*-*R1* decreased markedly during incubation with dexamethasone (0.25 ± 0.09 over baseline, *p* < 0.05).

Incubation with dexamethasone led to a decrease in *POMC* expression in most specimens (Fig. 3b). However, analysis of individual tumor cultures revealed that dexamethasone failed to inhibit *POMC* in some 20 % and even induced a paradoxical increase in *POMC* scattering of tumors (Fig. 3b). Of note, no difference in the proportion of high-dose dexamethasone responders in vivo was observed in the three groups (75, 55, and 80 % for specimens which inhibited, did not inhibit or presented a paradoxical *POMC* increase, respectively, N.S). Further, no difference according to sex, size of the adenoma and in vivo as well as in vitro CRH response status was observed (data not shown). As for baseline and CRH stimulation, *POMC* and ACTH response patterns were not correlated (*r* = −0.17, N.S.; Fig. 2c). Further, changes in *POMC* did not differ significantly according to the response to high-dose dexamethasone testing prior to surgery (1.00 ± 0.16 vs. 0.85 ± 0.13 over baseline, for in vivo high-dose dexamethasone responders and non-responders, respectively, N.S.) and an in vitro–in vivo concordance in the dexamethasone response pattern was observed in 43 % of patients only. Interestingly, glucocorticoid receptor mRNA was more abundant in specimens that failed to inhibit during incubation with dexamethasone (1.12 ± 0.15 vs. 0.62 ± 0.09 normalized expression for non-inhibitors and inhibitors, respectively, *p* < 0.01) and, indeed, was positively correlated with the ACTH response to dexamethasone (*r* = 0.61, *p* < 0.005). Much like *NR3C1* expression, both GR isoforms were slightly more abundant in specimens who failed to inhibit, although this did not reach statistical significance (GRα: 54.0 ± 15.7 vs. 24.0 ± 7.48 for non-inhibitors and inhibitors, respectively, N.S., GRß: 0.06 ± 0.004 vs. 0.02 ± 0.006 non-inhibitors and inhibitors respectively, N.S.). Dexamethasone also modulated GRα expression, roughly halving levels compared to baseline (0.64 ± 0.08, *p* < 0.05); no change in GRß during incubation with dexamethasone was recorded (0.92 ± 0.18 over baseline, N.S.).

## Discussion

The past few years have brought considerable progress in the knowledge of molecular changes in ACTH-secreting pituitary tumors, e.g., the involvement of epidermal growth factor [18] and orphan nuclear receptor TR4 [19], differing miRNA patterns [20], and, lately, also the presence of somatic mutations [21]. Lesser advances have been made as regards the response of tumoral corticotropes to the two main modulators of ACTH secretion, namely CRH and corticosteroids. In point of fact, a key feature of ACTH-secreting pituitary adenomas is that although ACTH secretion is autonomous, i.e., inappropriately high given excess cortisol levels, it remains sensitive to both CRH and strong negative feedback. In a clinical setting, responses to either CRH or steroid testing represent the framework for both diagnosis and assessment of treatment of Cushing’s disease [1, 11]; thus, a better understanding of the underlying mechanisms is needed.

Studies on human ACTH-secreting pituitary adenomas are constrained by the rarity and size of adenomatous specimens. In fact, past studies report on 20 specimens at most, more often <10, and frequently macroadenomas [22–24]. Thus, results may be biased by specimen selection; indeed, one study reported that adenomas are not responsive to CRH [24] but the same investigators showed that CRH does stimulate ACTH in subsequent experiments [25]. Further, a study on three ACTH-secreting adenomas reported a different hormonal phenotypic pattern in each specimen [26]. The present series on over 50 specimens represents the largest collection of adenomas reported up to now except for our previous series on 72 specimens [9] and provides representative data on this rare and complex disorder.

In this study, we analyzed the expression profiles of the three most important genes related to corticotrope secretion, namely *POMC*, CRH receptor subtype 1, and glucocorticoid receptors α and ß, both in basal condition and during incubation with CRH or dexamethasone. We can thus provide data on constitutive and modulated gene expression in adenomatous corticotropes, associated with tumor features and response to CRH and steroids.

First of all, we observed a considerable variability in *POMC* expression among corticotrope tumors. Other studies had evaluated POMC expression by in situ hybridization [7, 27] or RT-PCR [6, 8, 28] and reported on variable *POMC* signal intensity and expression levels. Of note, *POMC* signal density did not appear correlated with ACTH intensity assessed by immunohistochemistry [7, 27], a finding in keeping with our observation of absent correlation between *POMC* and ACTH. We observed divergent *POMC* and ACTH behavior in a given specimen both as regards constitutive as well as CRH- or dexamethasone-induced changes. Similar discrepancies have been reported during pharmacological challenges [29, 30] and support previously expressed hypotheses [7, 29] suggesting different regulatory mechanisms for *POMC* expression and ACTH secretion in corticotrope adenomas.

Along the same line, although *POMC* expression was greater in macroadenomas compared to microadenomas, no significant difference in spontaneous ACTH secretion according to tumor size could be detected in this as well as in larger series [9]. Impaired processing of POMC to ACTH has been observed in macroadenomas [31], a finding which may contribute to this result. Macroadenomas did not differ from microadenomas in any of the other parameters tested, e.g., *CRH*-*R1* and *NR3C1* expression, responsiveness to CRH or dexamethasone, supporting the concept that adenoma growth [32] does not proceed in parallel with hormonal activity.

Incubation with CRH brought about an increase in *POMC* in the majority of adenomatous specimens, in agreement with studies on some 10 ACTH-secreting adenomas [22, 33]. The present large series showed a highly individualized tumor response, from absent to lively. In fact, *POMC* increased from 20 to 500 % over baseline during CRH stimulation, even up to 14-fold in one adenoma. Interestingly, the magnitude of *POMC* increase was not correlated with CRH receptor abundance, a finding which dovetails with data obtained at immunohistochemistry showing that CRH-R1 staining density in adenomas is not proportional to the ACTH response to CRH [34]. Further, neither the in vivo nor the in vitro response to CRH was correlated with *CRH*-*R1* expression, suggesting that differences in receptor processing/signaling rather than synthesis [35] account for the tumoral corticotrope response.

The *CRH*-*R1* receptor was expressed at variable levels among specimens, confirming and extending findings reported on a small number of adenomas [5, 6, 36]. In detail, receptor binding studies had been performed in 5 adenomas, and considerable differences in the percentage of ^125^I-hCRH-labeled cells among specimens had been observed [37]. The *CRH*-*R1* gene itself reportedly was not mutated in ACTH-secreting pituitary adenomas [5]. *CRH*-*R1* expression in adenomatous specimens was modulated by CRH itself, with a median twofold increase. These results tally with data obtained by Northern blotting and binding studies showing increased receptor expression and continued responsiveness during incubation with CRH [38, 39]. In contrast, in normal anterior pituitaries, CRH down-regulates *CRH*-*R1* expression [38, 40] leading to receptor desensitization and attenuation of the response to CRH [35]. Thus, in adenomatous corticotropes, regulation of *CRH*-*R1* by CRH appears different from normal physiology, a finding which may contribute to the robust ACTH response to CRH in patients with Cushing’s disease.

Incubation with dexamethasone had diverging effects on *POMC* expression. In fact, both decrease in *POMC* as well as paradoxical increase in *POMC* expression were observed. Previous studies on a dozen of cases reported mean 20–40 % decreases in *POMC* during dexamethasone although some specimens failed to inhibit [22, 33]. The magnitude of our series allowed us to detect also paradoxical increases in *POMC* during incubation with dexamethasone, a finding which may be in line with paradoxical ACTH responses to other agents [26, 41] or to dexamethasone itself [9]. From a clinical viewpoint, paradoxical responses to high-dose dexamethasone testing have been observed in patients with Cushing’s disease [42, 43]. Experiments in normal rat anterior pituitaries consistently showed that dexamethasone reduces *POMC* expression [44]; thus paradoxical increases in *POMC*/ACTH represent a Cushing-specific derangement. Studies into corticosteroid modulation of *POMC* in Cushing’s disease have shown that Brg1 and HDAC2 protein expression in tumoral specimens differs to some extent according to dexamethasone sensitivity in vivo [3]. Further, additional contributors to the different dexamethasone response are variable 11ßHSD type 2 staining [4], *Hsp90* expression [45], and—possibly—a recently identified mediator of glucocorticoid resistance in corticotropes, *Cables1* [46].

As regards the glucocorticoid receptor, *NR3C1* expression was evenly distributed among tumoral specimens, without significant differences according to tumor size or surgical outcomes. Further, the GRα isoform was far more abundant than the truncated GRß isoform, as common in most tissues [47] and shown by others in corticotrope adenomas [28, 48, 49]. Studies in peripheral mononuclear cells as well as other cells have shown that levels of GR mRNA and protein are correlated with each other [47, 50], and thus gene expression provides useful clues for receptor abundance. Analysis of GR expression according to the pattern of response to dexamethasone in vitro revealed that *NR3C1* as well as GR isoforms were more abundant in specimens which failed to inhibit, a result which contrasts with Mu et al. [48] and Dahia et al. [49]. The former observed low levels of GRα in adenomas from 2 patients who failed to inhibit after 8 mg dexamethasone, and the latter failed to detect a correlation between GRα expression and response to 8 mg dexamethasone. Different experimental techniques, i.e., RT-PCR and quantification by ethidium bromide versus quantitative RT-PCR, the small number of samples as well as the mediocre correlation between in vivo and in vitro dexamethasone sensitivity [9] might explain the reported difference.

Lastly, ours is the first study to report on GR and CRH-R1 regulation by corticosteroids in human corticotrope adenomas. GRα expression was down-regulated by dexamethasone, as it occurs in most cells [51], at roughly the same potency and time course observed in other tissues [52, 53]. Likewise, regulation of *CRH*-*R1* by corticosteroids in corticotrope adenomas appeared similar to normal pituitaries; indeed, we observed halving of *CRH*-*R1* expression in corticotrope adenomas during incubation with dexamethasone, much like changes observed in normal rat anterior pituitary [40]. Altogether, it appears that glucocorticoid suppression of both GRα and *CRH*-*R1* is preserved in human corticotrope adenomas whereas glucocorticoid-mediated *POMC* modulation is variably altered. Divergent modulation of *NR3C1* and *POMC* by glucocorticoids has been shown to occur in AtT-20 cells, a murine tumoral corticotrope cell line [54], indicating different transcriptional interferences on these genes. It is worth recalling that the glucocorticoid receptor acts in concert with coactivators, chaperone proteins and chromatin-remodeling complexes in a tissue- and gene-specific manner [55]. The finding that glucocorticoids modulate *NR3C1*, *CRH*-*R1*, and *POMC* gene expression in a different fashion suggests that these effectors play a major role in corticotrope adenomas. Indeed, a recent study demonstrated that inhibition of HSP90 enhances dexamethasone-induced ACTH suppression in tumoral corticotropes [45].

In conclusion, our study reports on several novel features of human corticotrope adenomas. Foremost, *POMC* expression is variable among tumors and not correlated with ACTH levels, and, further, may exhibit a paradoxical increase during incubation with corticosteroids. It appears therefore that factors other than *POMC* synthesis account for ACTH release by tumoral cells and that *POMC* regulation by glucocorticoids is subject to unique derangements. Second, *CRH*-*R1* and *NR3C1* expression are not linked to the expected responses pointing towards a preeminent role of factors downstream to receptors themselves. These findings represent a starting point for future research into the mechanisms regulating ACTH secretion by human corticotrope adenomas.

## Electronic supplementary material

Below is the link to the electronic supplementary material.

Supplementary material 1 (DOC 20 kb)
