# Supplementary material for: Proopiomelanocortin, glucocorticoid, and CRH receptor expression in human ACTH-secreting pituitary adenomas
Source: Endocrine. 2016 May 24;55(3):853–60. doi: 10.1007/s12020-016-0990-x (PMC5316398; doi:10.1007/s12020-016-0990-x)
Supplement: Supplementary file 1 — Supplementary material 1 (DOC 20 kb) [file 12020_2016_990_MOESM1_ESM.doc]

**Supplementary information**

Number of specimens used for individual analyses:

- baseline *POMC* expression: 53 specimens

- baseline *CRH-R1* and *NR3C1* expression: 45 specimens

- *GRα* and *GRß* gene expression: 30 specimens

- CRH incubation: 47 specimens (13 for receptor expression)

- dexamethasone incubation: 45 specimens (13 for receptor expression)
